# Red fluorescence increases with depth in reef fishes, supporting a visual function, not UV protection

**DOI:** 10.1098/rspb.2014.1211

**Published:** 2014-09-07

**Authors:** Melissa G. Meadows, Nils Anthes, Sandra Dangelmayer, Magdy A. Alwany, Tobias Gerlach, Gregor Schulte, Dennis Sprenger, Jennifer Theobald, Nico K. Michiels

**Affiliations:** 1 Animal Evolutionary Ecology, Institution for Evolution and Ecology, Department of Biology, Faculty of Science, University of Tübingen, Auf der Morgenstelle 28, 72076 Tübingen, Germany; 2 Department of Marine Science, Faculty of Science, Suez Canal University, Ismailia, Egypt

**Keywords:** fluorescence, photoprotection, colour contrast, visual ecology, marine fish

## Abstract

Why do some marine fishes exhibit striking patterns of natural red fluorescence? In this study, we contrast two non-exclusive hypotheses: (i) that UV absorption by fluorescent pigments offers significant photoprotection in shallow water, where UV irradiance is strongest; and (ii) that red fluorescence enhances visual contrast at depths below −10 m, where most light in the ‘red’ 600–700 nm range has been absorbed. Whereas the photoprotection hypothesis predicts fluorescence to be stronger near the surface and weaker in deeper water, the visual contrast hypothesis predicts the opposite. We used fluorometry to measure red fluorescence brightness *in vivo* in individuals belonging to eight common small reef fish species with conspicuously red fluorescent eyes. Fluorescence was significantly brighter in specimens from the −20 m sites than in those from −5 m sites in six out of eight species. No difference was found in the remaining two. Our results support the visual contrast hypothesis. We discuss the possible roles fluorescence may play in fish visual ecology and highlight the possibility that fluorescent light emission from the eyes in particular may be used to detect cryptic prey.

## 1. Introduction

Natural fluorescent coloration is a striking and widespread feature of many marine organisms such as jellyfish [1,2], anemones [3] and corals [4], but also other marine invertebrates [5–9], and even vertebrates including marine birds [10,11] and many families of reef fishes [8,12]. Despite this ubiquitous pattern, it is still poorly understood why marine organisms fluoresce [5,13]. The functional hypothesis that has received the most support thus far proposes that fluorescent pigments provide protection against damaging UV in shallow water, as previously demonstrated for corals [5,14–19] and suggested for amphioxus [7] (see [4,13,20] for recent reviews of further functional hypotheses). This photoprotection hypothesis seems particularly relevant for sedentary, shallow-water organisms that rely on photosynthesis. It emphasizes wavelength-specific light absorption, a feature shared with other types of colour mechanism.

The second, alternative explanation derives from the fact that fluorescence emits photons at longer wavelengths following light absorption at shorter wavelengths [21]. Hence, by adding light to the long-wavelength range, fluorescence acts as an additive colour mechanism. This feature is unique to fluorescence and other forms of luminescence (e.g. chemi- or bioluminescence [21]). Virtually all animal colours, however, merely reflect or transmit light that is not absorbed. Consequently, they display a down-sampled subset of the ambient spectrum, which is why they are called subtraction colours. This applies to pigments as well as structural colours [22,23]. The key question is under what conditions additive fluorescent coloration can be significant for colour vision, given the evolutionary success of subtraction colours.

### (a) The role of fluorescence in colour vision

Natural luminescence, whether fluorescence or chemiluminescence, has one drawback: it is weak compared with the ambient sunlight in the same spectral range. As a consequence, the bioluminescent eyes of flashlight fish, for example, are only functional in twilight or darkness [24]. Such restrictions also apply to animal fluorescence. Although short-wavelength light is required to induce it, there should be little if any ambient light at the longer wavelengths where the fluorescent light is emitted. Hence, whenever the ambient spectrum covers the full visual spectrum—as is the case in terrestrial environments—fluorescence in animals may usually be insignificant relative to subtractive colours, explaining why the latter are usually the mechanism of choice [25] (see [10,26–31] for exceptions). This reasoning can be extended to clear shallow aquatic habitats [32,33]. We call these environments ‘euryspectral’ (i.e. they are characterized by an ambient spectrum that is so broad that it exceeds the visual spectrum of most animals at both ends of its range).

**Figure 1.**
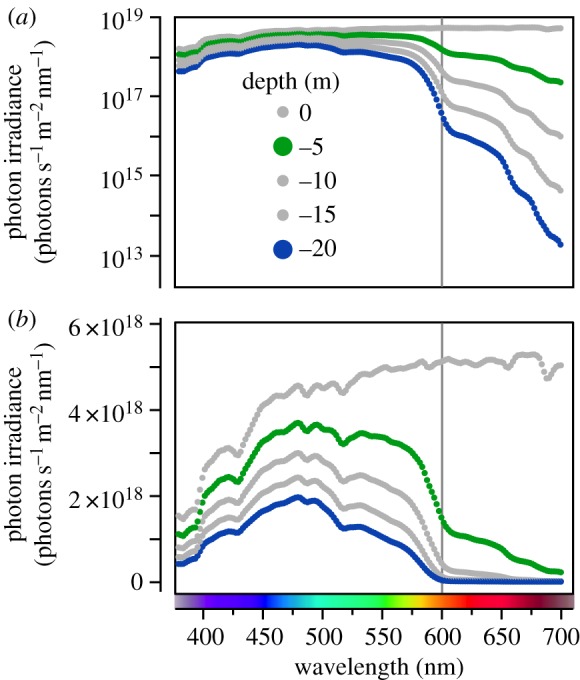
Vertically downwelling photon irradiance in the field expressed as a function of wavelength-based measurements taken at Sharm Fugani, Red Sea on a sunny day at noon in March 2013 (see the electronic supplementary material, S1 for methodological details). (*a*) *y*-axis log_10_-transformed, illustrating rapid light absorption in the 600–700 nm spectral range with increasing depth. (*b*) *y*-axis linear, providing a better resolution in the 380–600 nm range. This representation is also more intuitive concerning the proportional differences between the less than 600 nm and the greater than 600 nm ranges.

Conditions change in favour of fluorescence when descending further down the water column. In addition to getting darker, the spectrum quickly narrows in width because water absorbs long wavelengths (580–700 nm) particularly efficiently [23,34,35] (electronic supplementary material, S1; figure 1). Whereas total irradiance in the blue 450–500 nm range is balanced relative to that in the red 600–650 nm range just below the surface (blue/red = 0.856), this rapidly changes to a ratio of 186.4 at −20 m. The depth range in which the sunlight spectrum is narrower than the visual spectrum of many of its inhabitants will be called the ‘stenospectral’ zone hereafter. Near reefs the stenospectral zone starts between −10 and −25 m, depending on conditions such as waves, time of day, cloud cover and turbidity [36].

The stenospectral zone is ideal for a visual function of fluorescence [21]; while still offering sufficient light to induce fluorescence, there is little ambient light in the 580–700 nm emission range. As a consequence, even weak red fluorescence may become visible to an observer with the appropriate sensitivity. This is due to the way in which eyes perceive chromatic contrast; it depends on the *ratios* of cone photoreceptor types that are stimulated by light coming from an object compared with an adjacent object or background [25,37]. Hence, even when quantitatively weak, red fluorescent structures could produce a perceptible colour contrast against the cyan background of the stenospectral zone. In the shallow euryspectral zone, the contrast of a fluorescing structure would be insignificant against the broad spectral background.

Based on these considerations and as a non-exclusive alternative to the photoprotection hypothesis, the *visual contrast hypothesis* hypothesizes that fluorescence is used to generate patterns for long-wavelength vision in the stenospectral zone, as recently proposed for green fluorescence in midwater animals [13] and red fluorescence in barnacles [9].

### (b) Fluorescence in fish: photoprotection or visual contrast?

**Figure 2.**
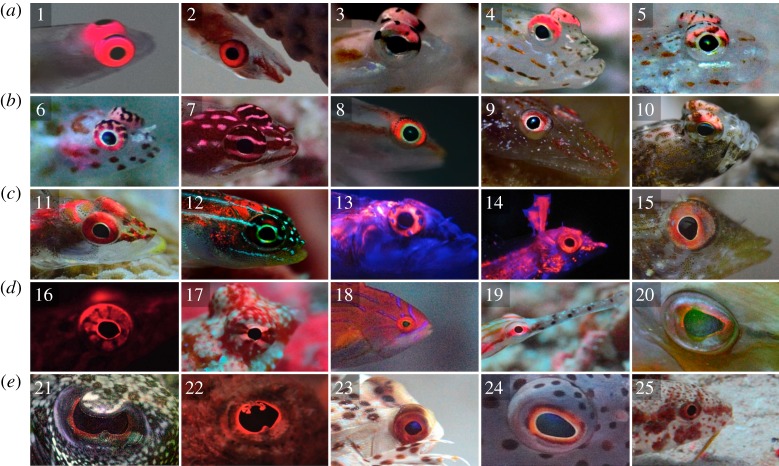
Fluorescent eyes of marine fish. Many red fluorescent marine fishes have their fluorescence concentrated in or near the eyes, illustrated here by 25 species from 12 fish families. (*a*,*b*) (Gobiidae—Gobies): 1, *Bryaninops natans*; 2, *Bryaninops loki*; 3, *Ctenogobiops feroculus*; 4, *Ctenogobiops maculosus*; 5, *Fusigobius melacron*; 6, *Eviota guttata*; 7, *Eviota zebrina*; 8, *Pleurosicya micheli*; 9, *Phyllogobius platycephalops*; 10, *Tomiyamichthys oni*. (*c*) (Tripterygiidae—Triplefins): 11, *Ucla xenogrammus*; 12, *Helcogramma striata*; 13, *Helcogramma steinitzi*; 14, *Enneapterygius pusillus*; 15, *Tripterygion delaisi*. (*d*,*e*) (other families): 16, *Lepadogaster candollei* (Gobiesocidae—Clingfishes); 17, *Synchiropus moyeri* (Callionymidae—Dragonets); 18, *Paracheilinus octotaenia* (Labridae—Wrasses); 19, *Corythoichthys schultzi* (Syngnathidae—Pipefish); 20, *Aulostomus chinensis* (Aulostomidae—Trumpetfishes); 21, *Bothus pantherinus* (Bothidae—Lefteye Flounders); 22, *Scorpaenopsis diabolus* (Scorpaenidae—Scorpionfish); 23, *Dactyloptena orientalis* (Dactylopteridae—Flying Gurnards); 24, *Paracirrhites forsteri* (Cirrhitidae–Hawkfishes); 25, *Upeneus tragula* (Mullidae—Goatfishes). Photographs were taken in the following regions: Mediterranean Sea in Croatia (15) and Corsica (16), Red Sea in Egypt (1, 4, 8, 13, 14, 18, 22, 24) and Indo-Pacific in Indonesia (Sulawesi and Raja Ampat, remaining pictures). Photographs 13, 14 and 16 were taken indoors; all others were taken while scuba diving. All photographs were taken with various red-enhancing filters. An additional blue light source was used for pictures 13, 14 and 16 (laboratory), 20 and 21 (field). All other pictures show fluorescence under natural light at depths below −15 m.

Recently, we described the presence of red fluorescence in several reef fish species [8] (see [12] for a further expansion). Many of these show a concentration of fluorescence in the head region and around the eyes, particularly in small fishes with an otherwise rather transparent body (figure 2). Tissues close to the eyes or the brain are particularly sensitive to photo-damage [38]. This is further substantiated by the fact that the ocular media of many reef fishes block UV [38,39]. All this indicates that the *photoprotection hypothesis* may be a valid explanation for fluorescence in fish—at least in species where fluorescence is located in sensitive structures. The *visual contrast hypothesis*, however, offers an attractive alternative for fish in the stenospectral zone. Many marine fishes possess photoreceptors with sensitivities extending into the long-wavelength part of the ambient spectrum, including families with many fluorescent representatives such as wrasses [40], pipefish [41] and gobies [8,42]. Hence, such species seem ideally adapted to use and perceive red fluorescence, as already suggested for the neon pygmy goby [8] and shown experimentally in the fairy wrasse *Cirrhilabrus solorensis* [43].

**Figure 3.**
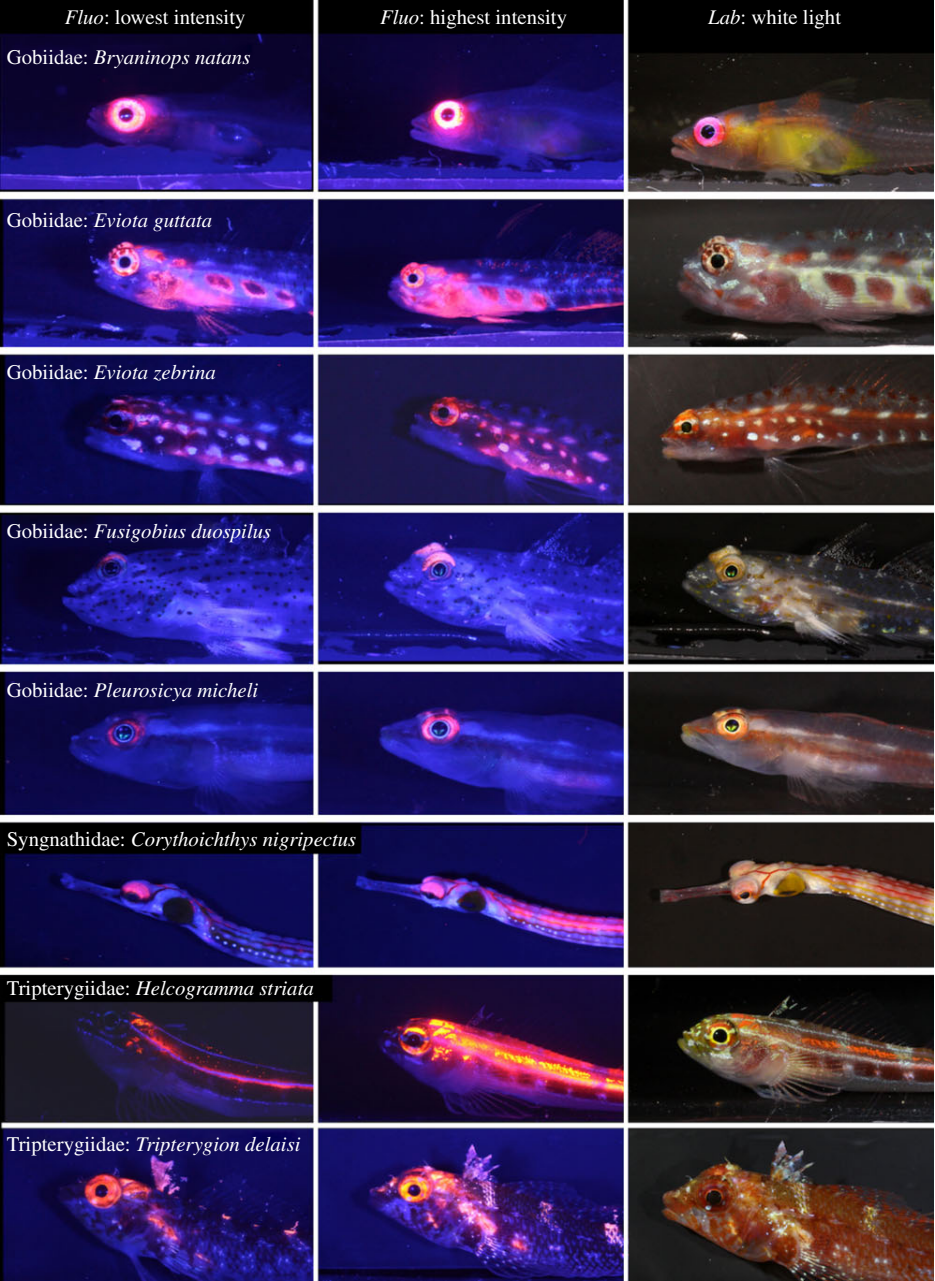
The eight study species under fluorescence and white light conditions. The first two columns were taken in the laboratory under standard fluorescence photography conditions, showing the individuals with the minimum (left column) and maximum (right column) fluorescence brightness among all sampled individuals. The third column shows individuals under standard white light conditions in the laboratory. Pictures were all taken under the same light conditions, explaining slight overexposure effects in species with strongly fluorescent eyes (triplefins *H. striata* and *T. delaisi*).

Although these hypotheses are non-exclusive, their relative effect can nevertheless be assessed because of the opposite predictions they make. Under the photoprotection hypothesis, fish should fluoresce more brightly in the euryspectral zone. Under the visual contrast hypothesis, fish are expected to fluoresce more brightly in the stenospectral zone. This allowed us to use a simple sampling design to competitively test which of the two hypotheses is more plausible: by measuring the brightness of red fluorescence in the eyes of eight different marine fish species from three fish families at −5 and −20 m (figure 3), we directly assessed whether fluorescence is linked more to the euryspectral or the stenospectral zone.

## 2. Material and methods

### (a) Focal fish species

Data were collected at sites in the Mediterranean Sea, Red Sea and Eastern Indian Ocean (see §2b). We selected species based on three criteria: (i) the presence of fluorescence in the iris, (ii) small size and benthic lifestyle to facilitate collection, and (iii) sufficient abundance at both sampling depths. Based on prior knowledge regarding the presence of red fluorescence and depth distribution [8] (N.K.M. 2007–2013, personal observation), we focused on eight species from three fish families.

Gobies (family Gobiidae) are the most species-rich marine fish family with correspondingly great diversification in terms of distribution, ecology and morphology [44]. They are mostly tropical and sub-tropical. The free-swimming redeye goby (*Bryaninops natans* [45]) usually forms groups from five to more than 50 individuals around compact *Acropora* coral heads, where they feed on plankton. The brightness of red fluorescence in *B. natans* irides is among the strongest recorded to date [8] (figure 3). The remaining four study species from this family (figure 3), the spotted pygmy goby *Eviota guttata* [46], the pygmy goby *Eviota zebrina* [46], Michel's ghost goby *Pleurosicya micheli* [47] and a sand goby, *Fusigobius* cf. *duospilus* [48], represent a species-rich guild of small bottom-dwelling predators that forage individually or in loose groups on benthic and planktonic prey. While *E. guttata*, *E. zebrina* and *P. micheli* primarily live on live hard corals (e.g. *Porites* boulders) and adjacent bare reef rock, *F.* cf. *duospilus* prefer the sediments at the reef base. All four species share reasonably strong fluorescence in the iris, with additional fluorescence on the head and upper flank in the two *Eviota* species (figure 3).

We included the black-breasted pipefish *Corythoichthys nigripectus* (cf. [49]) to represent the family Syngnathidae. This species inhabits sediment-rich reefs in coastal lagoons and seaward reefs, often in loose pairs or groups. Fluorescence is known from several members of this genus [8,12], with *C. nigripectus* displaying fluorescent patterns on the upper iris and to a variable extent along the upper body (figure 3).

Finally, triplefins (family Tripterygiidae) are mostly cryptobenthic, predatory blennioids with a worldwide distribution in tropical and temperate waters [50]. The black-faced blenny, *Tripterygion delaisi* [51] (figure 3), is a common inhabitant of rocky shores along the Eastern Atlantic and Mediterranean, where it forages mainly on small benthic invertebrates, often in shaded environments. The tropical striped triplefin, *Helcogramma striata* [52] (figure 3), is common on western Pacific coral reefs. It lives in small groups on hard coral and sponges, and feeds on zooplankton. As in many other triplefin species, both species exhibit strong fluorescence in and around the eye (and the upper flank in *H. striata*).

### (b) Sampling sites, permits and collection procedure

Field collection and spectrometry were conducted at three locations.

#### (i) Red Sea

All five goby species and the one pipefish species were collected from coral reefs in the bays of Sharm Fugani (Mangrove Bay) and Sharm Lassal (Utopia Beach), 20–30 km south of El Quseir, Egypt, in March 2013. Both locations offer protected reefs sloping down to −25 to −30 m. All fish were collected in the framework of a 3-year Memorandum of Understanding between the University of Tübingen represented by N.K.M. and the Suez Canal University represented by MAEA for the period 1 January 2013–31 December 2015.

#### (ii) Indo-pacific Ocean

The triplefin *H. striata* was collected at Hoga Island in the Wakatobi archipelago off the southeast Sulawesi coast, Indonesia in September 2011. Collection took place in the context of a general permit of Operation Wallacea to conduct scientific and educational projects on the reefs at Hoga (sampling registered accordingly). We sampled fish along the wall of an exposed reef (the ‘Pinnacle’) that slopes down to below −40 m.

#### (iii) Mediterranean Sea

The triplefin *T. delaisi* was collected in the Mediterranean Sea near the Station de Recherches Sous-marines et Océanographiques (Stareso) at Calvi, Corsica, France in June 2011. Samples were collected under the station's general sampling permit and registered accordingly. The site ‘La Bibliothèque’ is characterized by large granite boulders (greater than 5 m diameter) covered with algae and other encrusting organisms down to about −30 m.

### (c) Fish collection and maintenance

Fish were collected on scuba diving with hand nets after partially anaesthetizing individuals using clove oil where required (5% clove oil in 5% ethanol and 90% seawater [8]). Every dive focused on a single species. We usually reached our goal of approximately 10 individuals at each target depth on a single dive with two to four divers. We sampled at or below −20 m during the first half of the dive and at or above −5 m during the second half of the dive. After brief transportation in perforated 50 ml Falcon tubes or 1 l plastic bags (for *C. nigripectus*), fish were maintained in aerated containers at 24–26°C for 1–8 h. All individuals were measured on the collection day and released in their natural environment within 24 h. Sample size in *F.* cf. *duospilus* was initially 15 and 13 for the −5 and −20 m sites, respectively, but had to be reduced to 5 and 13 due to the inadvertent presence of 10 individuals of a sibling species (*F. neophytus*) in the −5 m sample, which we only discovered *a posteriori* when analysing the photographs.

### (d) Spectrometry and photographic documentation of fish fluorescence

We employed a standardized work flow in which each individual fish was (i) put in a plastic bag with a small amount of seawater, placed in ice water for about 1 min to tranquilize, (ii) placed in 1 cm of approximately 20°C seawater in a large glass Petri dish lined with non-fluorescent black cloth for spectrometric measurements for less than 5 min (details below), (iii) moved into 2 cm of approximately 20°C seawater in a photography chamber for standardized fluorescence pictures for less than 5 min (details below), and (iv) returned to a recovery tank with 20 l of aerated seawater at room temperature.

Spectrometric measurements were taken with an Ocean Optics QE65000 spectrometer for fluorescence and a bifurcated OceanOptics QR600–7-UV125BX fibre optics cable with a single saltwater proof tip in which six peripheral bundles of glass fibres emit the excitation light and one central bundle of glass fibres collects the emitted light. Excitation light was generated using a green laser (ThorLabs CPS532, a 532 nm laser diode module with an AHF narrow-band laser clean-up filter ZET 532/10) and guided into the illumination arm of the bifurcated fibre. With this excitation illumination, the fluorescent signal is maximized when the submerged probe is held at a distance of 4.5–5 mm. At this distance, the viewing angle of the central, light-accepting fibre has a diameter of 1.51–1.67 mm (area 1.79–2.19 mm^2^). The fibre guiding the accepted light to the spectrometer included a filter holder with a Semrock EdgeBasic 532R-25 long-pass filter to eliminate reflected laser light.

Each new fish measurement series included a control measurement of a Labsphere Spectralon Fluorescence Standard (type USFS-336–010) to check for fluctuations in measurement sensitivity. Spectrometer integration times were usually 800 ms, but adjusted to shorter integration times when emission intensities exceeded the dynamic range of the spectrometer (e.g. in *B. natans*). All final measurements were uniformly expressed as counts ms^−1^ nm^−1^. The basic set-up at Hoga Island (for *H. striata*) was similar, but used a bifurcated Avantes 7UV200 fibre optics cable and generated excitation light with a different green 532 nm laser pointer (Conrad, part number 776301–62). The set-up at Stareso (for *T. delaisi*) used another green laser pointer (Z-Bolt Scuba-1/Dive Laser) as excitation source, but was otherwise identical. As a consequence of these differences between set-ups, the readings for the three sites cannot be compared quantitatively. However, because our goal was to examine differences in fluorescence brightness within species, this limitation does not affect the interpretation of our results.

For the actual fluorescence measurements, the tip of the spectrometer probe was handheld by one person, pointing at the fish with the tip submerged at the optimal focal distance (approx. 0.5 cm) and an angle of approximately 45° to the fish held in an upright position in the Petri dish. Both eyes were measured. The emission signal fluctuations inherent to this type of spectrometry were mitigated by repeating measurements up to 10 times per individual fish eye. To exclude sequence or handling effects, fish were measured in a randomized order with respect to depth of origin, with the person doing the measurements blind for fish origin. All measurements were taken in a dark room, only dimly illuminated with 450 nm LEDs (invisible to the spectrometer set-up).

Fish were photographed in a matte black chamber 2 cm deep, 15 cm wide and 15 cm high, with a thin glass front (1 mm). A ruler and a Labsphere spectralon white and red standard were positioned at the front. Pictures were taken with a Canon EOS 7D camera with a 60 mm macro lens through a LEE orange (#105) filter. For fluorescence imaging, two blue LED Hartenberger Mini Compact LCD with 7 × 3.5 W 450 nm LEDs illuminated the chamber in a 45° angle from the left and right. A Thorlabs FD2C subtractive dichroic color bandpass filter on each torch suppressed long wavelengths in the excitation light. Regular pictures were taken using a Princeton Tec Torrent LED torch. Fish size (length in millimetre) was measured from the caudal peduncle to the tip of the mouth using ImageJ (v. 1.47, http://rsb.info.nih.gov/nih-image/index.html).

### (e) Statistical analysis

**Figure 4.**
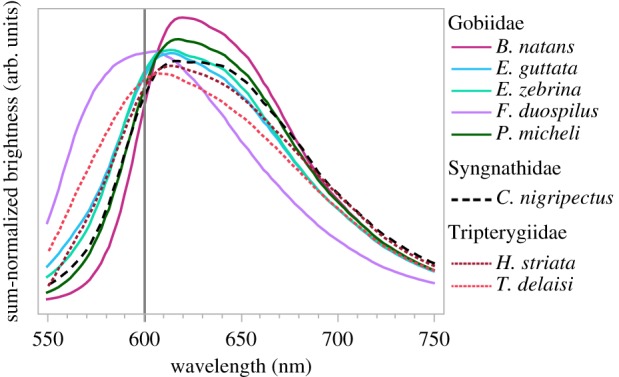
Fluorescent emission spectra of the eight study species averaged and sum-normalized across all measured individuals using the maximum curve for each individual. All species show a peak emission in the spectral range where absorption by water increases rapidly (cf. figure 1).

We calculated fluorescent emission brightness as the integrated area under the emission curve (counts ms^−1^ nm^−1^; ‘total brightness’ [53,54]) with the highest fluorescent signal from either of the two eyes for each individual fish, limited to the focal ‘red’ emission range between 580 and 750 nm (figure 4). Although ‘counts’ are closely and linearly related to ‘quanta’ (the Ocean Optics QE65000 has a 90% quantum efficiency in the target emission range), we did not actually measure the excitation curves and quantum yields of the fluorescent pigments and therefore need to treat these measurement as ‘arbitrary units’, which are useful for relative comparisons within the same dataset, but not between datasets obtained with different (artificial or natural) excitation sources. Given that these measurements tended to show left-skewed distributions with inhomogeneous variances between depths, we performed our analysis using log_10_-transformed values. Alternative measures of fluorescence brightness (maximum or mean peak emission per fish, or mean integrated total emission per fish) yielded qualitatively identical results.

Because of the differences in ecology and behaviour of the species tested, it is possible that a visual contrast or photoprotection function apply differently to each of them. For this reason, we refrained from carrying out an overall statistical analysis, but analysed the data for each species independently. Our prime analysis compares fluorescent brightness between individuals caught in shallow (−5 m) and deep (−20 m) water for each species. In addition, our analysis took into account individual body length as a covariate (ANCOVA). In five species (*B. natans, C. nigripectus, E. guttata, E. zebrina* and *F.* cf. *duospilus*), individuals from shallow water tended to be larger on average than those from deeper water (Welch's *t*-test, all *p* < 0.088) with the reverse pattern in *T. delaisi* (*p* = 0.026) and no difference in *P. micheli* and *H. striata* (all *p* > 0.42). This non-independence between our main factor (depth) and the covariate (body length), however, did not qualitatively affect our results: first, fluorescence brightness was independent of body length in seven species (linear regression, all *p* > 0.11) and only showed slight positive covariation in *H. striata* (adj. *R*² = 0.139, *F*_1,14_ = 3.42, *p* = 0.086). Second, we found no heterogeneity in covariate regression slopes between the two depths (ANCOVA, interaction body length × depth, all *p* > 0.54) except for *H. striata* (*p* = 0.03). Finally, visual data inspection (electronic supplementary material, figure S2) reassures that the reported depth effects on fluorescence brightness are not confounded by covariation with body length within the body size range of our measured fish. All our findings are robust to inclusion or exclusion of body length as a covariate, as well as to alternative non-parametric testing. Statistical analyses were performed in R (v. 3.0.1, R Development Core Team).

## 3. Results

The irides of all eight species sampled showed a fluorescence emission peak in the range of 600–620 nm (figure 4). There was conspicuous individual variation in fluorescence brightness in some, but not all species (figure 3).

**Figure 5.**
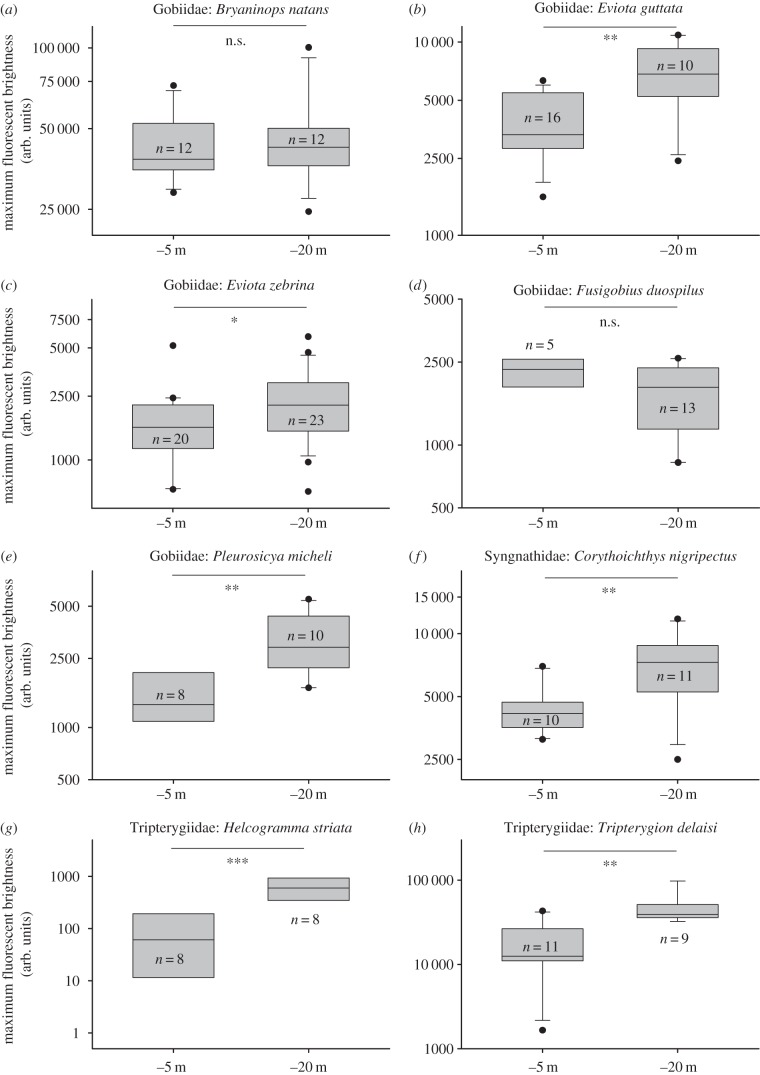
The effect of depth on fluorescence brightness expressed in arbitrary units (see Material and methods) at the −5 and −20 m target depths. n.s. = *p* > 0.05, *p < 0.05, **p < 0.01, ***p < 0.001. See table 1 for details of statistical analysis. Note different scaling on the *y*-axes.

In six out of eight species, fluorescence was significantly brighter at −20 m than at −5 m (table 1 and figure 5). The effect was particularly strong in the gobies *E. guttata* and *P. micheli*, the two triplefins *H. striata* and *T. delaisi*, and the pipefish *C. nigripectus.* It was less pronounced but still significant in the goby *E. zebrina*. No effect was found in the two gobies *B. natans* and *F*. cf. *duospilus*.

**Table 1.** Analysis of covariance comparing fluorescence brightness (log_10_-transformed counts ms^−1^; see Material and methods) between the −5 and −20 m sampling depths, including body length as a covariate. Bold *p*-values highlight statistically significant effects at *α* = 0.05.

family	species	factor	d.f.	*t*	*p*
Gobiidae	*B. natans*		Model adj. *R*² = 0.04, *F* = 1.51, *p* = 0.245		
		depth	1	−1.27	0.220
		body length	1	1.60	0.130
		error	21		
Gobiidae	*E. guttata*		Model adj. *R*² = 0.30, *F* = 6.25, *p* = 0.0068		
		depth	1	−3.49	**0.002**
		body length	1	0.61	0.547
		error	23		
Gobiidae	*E. zebrina*		Model adj. *R*² = 0.06, *F* = 2.39, *p* = 0.105		
		depth	1	−2.18	**0.035**
		body length	1	0.62	0.540
		error	40		
Gobiidae	*F.* cf. *duospilus*		Model adj. *R*² = −0.008, *F* = 0.93, *p* = 0.416		
		depth	1	1.19	0.250
		body length	1	0.21	0.840
		error	15		
Gobiidae	*P. micheli*		Model adj. *R*² = 0.43, *F* = 7.49, *p* = 0.006		
		depth	1	−3.81	**0.0017**
		body length	1	−0.31	0.7619
		error	15		
Syngnathidae	*C. nigripectus*		Model adj. *R*² = 0.37, *F* = 6.94, *p* = 0.0058		
		depth	1	−3.47	**0.0027**
		body length	1	1.84	0.0825
		error	18		
Tripterygiidae	*H. striata*		Model adj. *R*² = 0.514, *F* = 8.95, *p* = 0.0036		
		depth	1	−4.21	**0.001**
		body length	1	−0.54	0.600
		error	13		
Tripterygiidae	*T. delaisi*		Model adj. *R*² = 0.40, *F* = 7.28, *p* = 0.0052		
		depth	1	−2.9	**0.010**
		body length	1	0.82	0.420
		error	17

Body size did not contribute significantly to variation in fluorescence brightness in seven species, and marginally so in only one, namely *C. nigripectus* (see Material and methods; electronic supplementary material, S2).

## 4. Discussion

Fluorescence brightness differed significantly between the euryspectral and stenospectral sampling zones for six out of eight species, indicating that fluorescence is adjusted to depth. In all six species, the fluorescing irides were significantly brighter at greater depth, and none of the species examined showed the opposite pattern. Although we used species from three fish families and seven genera, fluorescent peak emission was very similar at 600–620 nm in all eight species. Our findings are consistent with the hypothesis that six species of fish studied here have red fluorescence mainly for visual, contrast-enhancing functions. This does not imply that photoprotection is irrelevant, but it is likely to be of secondary importance in the species sampled here. Whether a corresponding depth effect is absent in *B. natans* and *F.* cf. *duospilus* because both mechanisms act simultaneously or because those fish lack depth-based adaptations is currently not clear. The role of fluorescent pigments for photoprotection has previously been investigated for corals [16–19], but, to our knowledge, not for vertebrates. We now provide indirect evidence that photoprotection is probably not the primary function in at least some marine fish.

We suspect that the observed difference between the two depths involves differences in the number of melanophores covering the iris, the number of fluorescent chromatophores and/or the concentration of fluorescent pigment within the fluorescent chromatophores. Because we did not correct for the size of the fluorescent patch, we cannot exclude that fluorescent patch size may also have contributed to the observed effect. The differences in fluorescence brightness could originate from phenotypic plasticity during development or in the adult stage, or due to local genetic adaptation. *Tripterygion delaisi* is known to exhibit high levels of self-recruitment [55] and population genetic sub-structure [56], but only when its rocky shore habitats are isolated by large discontinuities of sand or deep water at a scale of kilometres. While quantifications of depth-related population sub-structure are missing, this renders small-scale local adaptation as known for other fish [57] at least unlikely. In the adult stage, however, all investigated fish inhabit spatially limited, benthic home ranges, with adult dispersal of *T. delaisi* estimated at just a few dozen metres [56]. The resultant depth-range fidelity may offer sufficient time to phenotypically adjust the machinery controlling iris fluorescence to the local light conditions. The contributions of plasticity and genetic differentiation to observed differences in fluorescence are subjects of current research.

The suggestion that fluorescence has a visual function in marine fishes fits well with the recent discovery that males of the fairy wrasse *Cirrhilabrus solorensis* respond to the deep red fluorescence typical of this species in a mirror image stimuli experiment [43]. It also adds to a small but growing collection of corresponding case studies in other animal systems. Fluorescence has been proposed to have a signalling function in mantis shrimps [6], jumping spiders [26] and budgerigars [28]. In deep-sea dragonfish, it is used to transform green bioluminescent light into red light [58]. We expect that visual functions of fluorescence may be widespread in animals with well-developed colour vision living under spectrally skewed environments.

Our study does not answer the underlying question of why reef fish may benefit from increasing visual contrast using red fluorescence. Observations on many marine fish species show that red fluorescence can be present on many parts of the body and in a variety of patterns, suggesting visual functions in intra- and interspecific signalling, camouflage or warning [8,12]. Fluorescence around the eyes is often found in small, highly cryptic, benthic, predatory fish [8] (figures 2 and 3), suggesting a functional link. Bruce [59] proposed that fluorescence around the eyes may not act as a signal to an observer, but as an active light source used by the sender. Being close to the pupil makes fluorescent irides ideally positioned to generate reflections in the eyes of cryptic prey. Under stenospectral conditions, such reflections generated using red fluorescence would contrast strongly with the cyan visual background. This idea has striking analogies with a similar mechanism described for nocturnal, bioluminescent fish [24] and deserves more attention in future research.

## 5. Conclusion

Fluorescence brightness increased with depth in six out of eight marine fish species. This is opposite to the pattern expected if long-wavelength fluorescence were to primarily serve photoprotection. Our data are, however, consistent with the alternative hypothesis, which states that fluorescence can serve a visual contrast function when the wavelengths emitted by fluorescence are rare or absent from the ambient light. Visual contrast enhancement offers an intriguing new adaptive function for fluorescent pigments in marine environments, which calls for investigations of the physical properties, perceptive abilities and behavioural consequences of signalling using locally rare colour hues.

## Supplementary Material

Estimating the ambient light along a depth gradient at a Red Sea coral reef

## Supplementary Material

Co-variation between fluorescence brightness and individual body length

## Supplementary Material

DATA: Estimated ambient attenuation coefficients

## Supplementary Material

DATA: Estimated ambient spectra at depth

## Supplementary Material

DATA: Spectral data for all fish specimens at both depths

## Data accessibility

All supporting data are available in the electronic supplementary materials.
