# Supplementary material for: Red fluorescence increases with depth in reef fishes, supporting a visual function, not UV protection
Source: Proc Biol Sci. 2014 Sep 7;281(1790):20141211. doi: 10.1098/rspb.2014.1211 (PMC4123709; doi:10.1098/rspb.2014.1211)
Supplement: Estimating the ambient light along a depth gradient at a Red Sea coral reef [file rspb20141211supp1.docx]

## **Meadows et al. – Electronic Supplementary Information 1**

## Estimating the ambient light along a depth gradient at a Red Sea coral reef

Spectral changes along reefs have been documented by others (e.g. [1,2]). Here, we present detailed data for our main study site Sharm Fugani, Red Sea, along with practical details on how to measure and process such data. We measured photon irradiance using a calibrated PhotoResearch PR-670 radiometer with cosine integrator PhotoResearch CR-670-Straight in a custom-made underwater housing ([www.UK-Germany.com](http://www.UK-Germany.com)). Two tubular levels, a floating compass, and an electronic depth gage were attached to the housing to facilitate hand-held positioning in the water column. Prior to underwater deployment, changes in spectral intensity and shape due to the optical port of the UW-housing were measured to correct the raw data. We measured vector irradiance, i.e. all light energy striking a flat surface from a hemisphere, in W·m^-2^·nm^-1^ from 380‑780 nm. We took measurements along two depth gradients at the reef along the northern edge of Sharm Fugani, El Quseir, Red Sea, Egypt. The first series was measured on 9 March 2013 ca. 1 m away from the reef slope while aiming horizontally towards the substrate, which faces SSE. Data were collected in 1 m intervals from just below the surface (= *I_0_*) to -15 m. Two more series were measured during a single dive on 11 March 2013 in open water (ca. 100 m south of the reef edge) along a vertical buoy line anchored in a flat sandy area with patches of seagrass in –30 m. Measurements were taken in 2 m depth intervals from just below the surface (= *I_0_*) to –24 m. During the first, descending series, the radiometer was pointed vertically upwards to collect downwelling light. During the second, ascending series, it was pointed horizontally into the blue water in a southern direction to collect sidewelling light. Care was taken to keep the operator and his dive buddy out of the hemispheric field of view of the cosine integrator. Both dives took place on a bright, sunny day at approximately solar noon, meaning that the change in solar irradiance between the start and end of the dives was negligible. A weak wind from the North caused ripples on the surface on both measurement days. At depths below -10 to -14 m depth, the measurements became noisy in the long wavelength range (> 600 nm). We therefore used irradiance data down to -10 m to calculate diffuse attenuation coefficients (*c*) for each wavelength using this formula:

$c=-\ln\left(\frac{I_{d+1}}{I_{d}}\right)$ (1)

*c* includes he combined affects of scattering and absorbance, both of which can be described with the same function [3]. *I_d+1_* and *I_d_* represent the amount of light measured at depth *d* and *d* + 1. In cases like ours where measurements have not taken place over 1 m intervals, (1) can be generalized as follows:

 (2)

with *z* = path length between two subsequent measurements. In order to calculate *c*, we first determined the mean value of *I_d_*_+_*_z_*/*I_d_* for each depth *d* for which measurements were available (Figure S1). These values were used to calculate *I_d_* at depth *d* at each wavelength*λ* as follows [3] (see Figure 1, main text):

$I_{d}=I_{0}e^{-cd}$ (3)

with

*I_d_* = light arriving at depth *d*

*I_0_* = light just below the surface

*d* = absolute vertical distance from the surface in m (positive value)

*c* = extinction coefficient per meter of water

These measurements should be seen as a pragmatic “black box” assessment of the combined effects of absorption, complex scattering and reef (chlorophyll) fluorescence (Figure S1). They ignore the details of the underlying physical processes and some depth-dependent variation in scattering and absorption caused by plankton or the vicinity to the surface (e.g. flicker and scatter shading, see Figure S1). Yet, it is an indispensible tool to interpolate and extrapolate (to a moderate extent) spectra at depths for which measurements are hard to obtain (e.g. the long wavelength range > 600 nm).

**References**

1. Chiao, C. C., Cronin, T. W. & Osorio, D. 2000 Color signals in natural scenes: characteristics of reflectance spectra and effects of natural illuminants. *J. Opt. Soc. Am. A. Opt. Image Sci. Vis.* **17**, 218–224.

2. Maritorena, S. & Guillocheau, N. 1996 Optical properties of water and spectral light absorption by living and non-living particles and by yellow substances in coral reef waters of French Polynesia. *Mar. Ecol. Prog. Ser.* **131**, 245–255.

3. Johnsen, S. 2012 *The optics of life: a biologist’s guide to light in nature*. Princeton: Princeton University Press.

**Figure ESM 1.1:** Attenuation coefficients expressing the proportional loss of light per meter in the 380-700 nm spectral range based on irradiance measurements along a depth gradient from just below the surface down to –14 m for light coming down vertically (*Downwelling*), from the substrate (*Reef*) and laterally from the open water (*Sidewelling*). The dip in the *Reef* curve at around 680 nm does not indicate reduced absorbance at that wavelength, but is the additive affect of chlorophyll fluorescence from the reef substrate. The slightly negative values for the *Sidewelling* (blue) curve in the 380-550 nm range is due to an initial increase in scatter when moving down from just below the surface. It reaches its maximum at around -4 m from which it starts to decrease again slowly. Only at -14 m did the sidewelling light reach a light level that was identical to that just below the surface (0 m). We think this effect can be attributed to flicker caused by ripples on the water as well as reduced lateral scatter just below the surface.

**Figure ESM 1.2: Photon irradiance in the field** expressed as a function of wavelength. Irradiance measurements are based on three sets of measurements taken at Sharm Fugani, Red Sea on a sunny day at noon in March 2013 (see Electromic Supplementary Material 1). **Top**: Looking vertically upwards (Downwelling). **Middle**: looking horizontally towards the reef (in northern direction at a distance of 2 m) (Reef). **Bottom**: looking horizontally into the open water in a southern direction (Sidewelling). Y-axes are log_10_ transformed, indicating orders of magnitude. The vertical line at 600 nm separates the less-absorbed part of the spectrum (to the left) from the more strongly absorbed part (to the right).
