# Supplementary material for: Red fluorescence increases with depth in reef fishes, supporting a visual function, not UV protection
Source: Proc Biol Sci. 2014 Sep 7;281(1790):20141211. doi: 10.1098/rspb.2014.1211 (PMC4123709; doi:10.1098/rspb.2014.1211)
Supplement: Co-variation between fluorescence brightness and individual body length [file rspb20141211supp2.docx]

## **Meadows et al. – Electronic Supplementary Information 2**

**Figure ESM 2**: Co-variation between fluorescence brightness and individual body length of the eight study species. Graphs show regression lines and their 95% confidence intervals split for fish caught at -5 m (open circles, broken line, pale grey areas) and -20 m (filled circles, solid line, dark grey areas) depths. Note log-scales on Y-axes.
